# Case Report: Cardiac myxomas and Carney complex: a case of recurrent embolic strokes and intracranial tumor growth

**DOI:** 10.3389/fonc.2025.1605692

**Published:** 2025-06-26

**Authors:** Aliny W. Kuhn, Antonio M. Lerario, Alice N. R. Morais, Ricardo F. Iglesio, Félix J. A. Ramires, Ligia C. A. Maluf, Ana O. Hoff, Ana C. Latronico, Berenice B. Mendonça, Madson Q. Almeida, Maria C. B. V. Fragoso

**Affiliations:** ^1^ Adrenal Unit, Hormones and Molecular Genetics Laboratory (LIM/42), Division of Endocrinology and Metabolism, Hospital das Clínicas, Faculty of Medicine, University of São Paulo, São Paulo, Brazil; ^2^ Department of Internal Medicine, Division of Metabolism, Endocrinology, and Diabetes, University of Michigan, Ann Arbor, MI, United States; ^3^ Division of Oncology, São Paulo State Cancer Institute, University of São Paulo, São Paulo, Brazil; ^4^ Division of Neurosurgery Oncology, São Paulo State Cancer Institute, University of São Paulo, São Paulo, Brazil; ^5^ Division of Cardiology, Hospital das Clínicas, Faculty of Medicine, University of São Paulo, São Paulo, Brazil; ^6^ Division of Radiotherapy, São Paulo State Cancer Institute, University of São Paulo, São Paulo, Brazil; ^7^ Division of Endocrinology, São Paulo State Cancer Institute, University of São Paulo, São Paulo, Brazil

**Keywords:** brain tumor, emboli, cardiac myxoma, Carney complex, case report

## Abstract

Cardiac myxomas, though rare, are the most common benign cardiac tumors and may be associated with Carney Complex (CNC). Patients with CNC are at increased risk of developing recurrent myxomas, which can lead to severe complications. We report a case of a 46-year-old woman with CNC and recurrent cardiac myxomas who developed multiple embolic strokes and cerebral aneurysms. Following two hemorrhagic strokes, neuroimaging and biopsy revealed a well-differentiated myxoid neoplasm in the brain parenchyma. Genetic analysis revealed a germline pathogenic *PRKAR1A* variant, along with loss of heterozygosity (LOH) at chromosome 17q24.2 in the cardiac myxoma, but not in the brain lesion. This case challenges the conventional understanding of cardiac myxomas as strictly benign, suggesting they may exceptionally exhibit distant proliferative behavior, likely through mechanical dissemination and subsequent growth in the brain. Although embolic events are common in cardiac myxomas, the capacity of tumor cells to implant and proliferate in extracardiac sites remains poorly understood. Our findings underscore the importance of maintaining a high index of suspicion for neurological complications in patients with cardiac myxomas, particularly in the setting of CNC. Further investigation is essential to elucidate the mechanisms driving this behavior and to optimize management strategies in similar cases.

## 1 Introduction

Primary cardiac tumors are rare, and cardiac myxomas represent the most common benign subtype (1, 2). They may arise sporadically or in association with Carney Complex (CNC), an autosomal dominant syndrome marked by a wide range of benign and malignant neoplasms affecting the heart, skin, endocrine organs, and nervous system (3). Given its multisystem involvement and high risk of tumor recurrence, Carney Complex mandates lifelong, protocol-driven surveillance in line with established clinical guidelines and consensus-based recommendations (4, 5).

CNC is diagnosed based on established clinical and molecular criteria (**Table 1**), with germline pathogenic variants in the *PRKAR1A*, which encodes the type 1A regulatory subunit of the cAMP-dependent protein kinase, being the most frequently identified genetic cause (4, 5). Around 20%–40% of individuals with CNC develop cardiac myxomas (6). Despite being histologically benign, cardiac myxomas can be clinically aggressive, with a propensity for complications such as fragmentation, embolization, local invasion, and regrowth. In rare cases, metastasis-like dissemination to distant sites, including the pelvis, spinal cord, sternum, and brain, has been reported (7). This report describes a rare and complex case of CNC with recurrent cardiac myxomas, complicated by cerebral aneurysms and a myxomatous brain lesion. It raises important questions about the potential for tumor cells to migrate and grow beyond the heart.

**Table 1:** Major and supplemental criteria for CNC.

Major criteria	
1.	Spotty skin pigmentation with a typical distribution (lips, conjunctiva and inner or outer canthi, vaginal and penile mucosa)*
2.	Myxoma (cutaneous and mucosal)*
3.	Cardiac myxoma*
4.	Breast myxomatosis* or fat-suppressed MRI findings suggestive of this diagnosis
5.	PPNAD* or increased urinary cortisol after high-dose dexamethasone administration
6.	Acromegaly due to GH-producing adenoma*
7.	Thyroid carcinoma* or multiple hypoechoic nodules on thyroid ultrasonography, in a young patient
8.	Psammomatous melanotic schwannoma*
9.	Blue nevus, epithelioid blue nevus (multiple)*
10.	Breast ductal adenoma (multiple)*
11.	Osteochondromyxoma*
12.	LCCST* or characteristic calcification on testicular ultrasonography, in a young patient

Supplemental criteria	
1.	Affected first-degree relative
2.	Inactivating pathogenic variant in the *PRKAR1A* gene

*With histological confirmation.The diagnosis is confirmed if the patient meets at least two diagnostic criteria or one diagnostic criterion plus one supplemental criterion (4, 5).

## 2 Case description

A 46-year-old female patient, previously diagnosed with Carney Complex (CNC; OMIM# 160980), presented with a history of adrenocorticotropic hormone (ACTH)-independent Cushing’s syndrome, supported by histology as primary pigmented micronodular adrenal disease (PPNAD). Additional clinical features included labial lentigines, multiple cutaneous myxomas, thyroid nodules, mammary microcalcifications, elevated serum levels of growth hormone and insulin-like growth factor 1 (IGF-1), and a recurrent left atrial myxoma. Genetic testing revealed a germline pathogenic *PRKAR1A* variant (c.491_492delTG, p.Val164Aspfs*5; rs281864790 in heterozygosity), which has been linked to an increased risk of cardiac myxomas, lentigines, and thyroid tumors compared to other genotypes (8).

Prior to the current clinical course, the patient had undergone two cardiac surgeries to remove myxomas. The first surgery addressed a right atrial tumor measuring 6.7 × 4.5 cm. Seven years later, imaging reveled a new myxoma in the oval fossa of the left interatrial septum (3.5 × 2.9 × 1.3 cm), along with recurrence of the previously excised tumor, now reduced to 1.7 × 1.1 × 1.1 cm.

**Figure 1:**
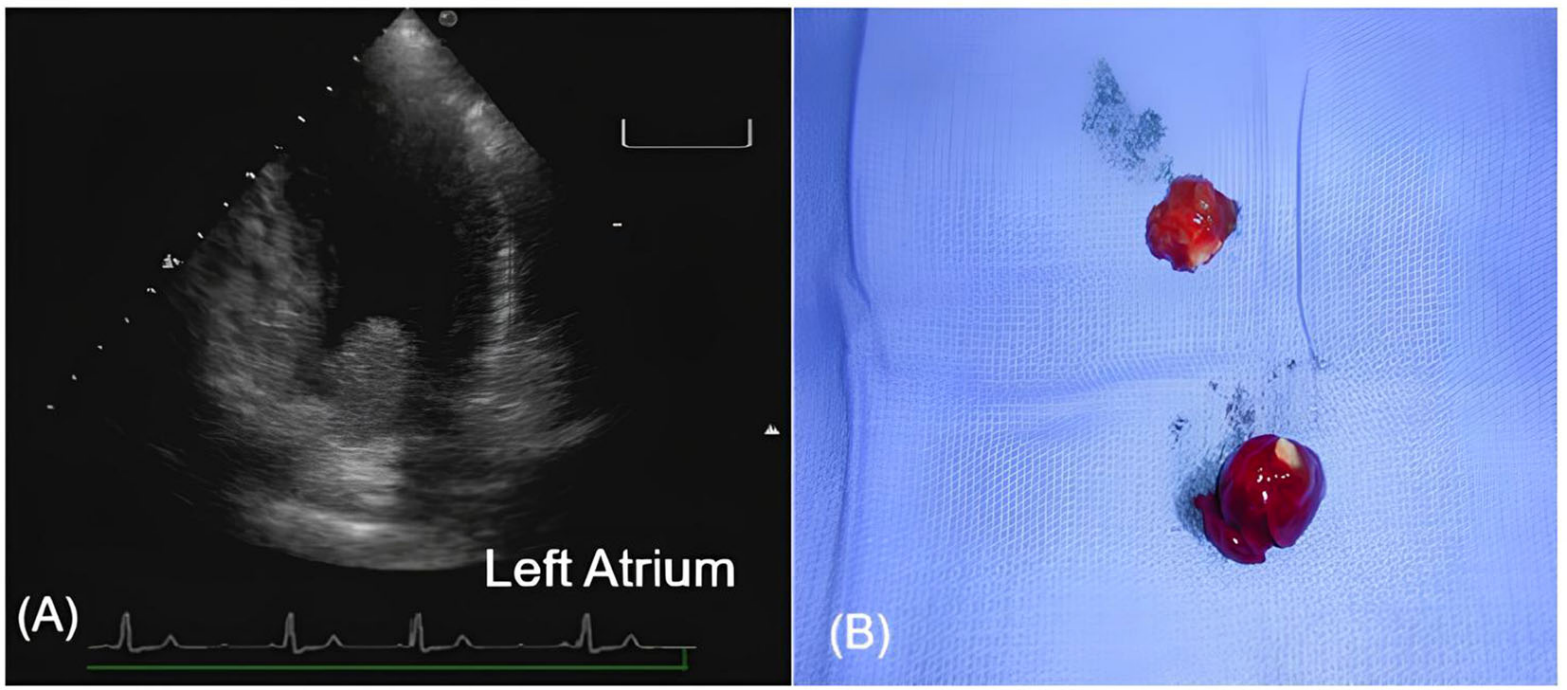
Recurrent left atrial myxoma originating from the interatrial septum. **(A)** Transthoracic echocardiography (apical four-chamber view) showing a mobile mass in the left atrium attached to the interatrial septum, measuring approximately 3.3 × 2.5 x 1.5 cm. **(B)** Gross pathology of the excised myxoma, showing a lobulated gelatinous tumor consistent with typical myxomatous appearance.

Seven months before the current presentation, the patient experienced sudden motor aphasia. Brain CT revealed an ischemic stroke in the left middle cerebral artery territory. By that time, recurrence of the myxoma had already been detected, but surgical resection was postponed owing to restrictions related to the COVID-19 pandemic. Antithrombotic therapy was withheld, as the presumed underlying mechanism was embolization secondary to tumor fragmentation. Several months later, she suffered a second stroke involving multiple vascular territories, accompanied by cerebral and cerebellar microhemorrhages. The atrial myxoma (now measuring 3.3 × 2.5 × 1.5 cm, **Figure 1**) was considered the most likely source of these embolic events. Importantly, no clinical signs or symptoms suggestive of systemic inflammatory syndrome were observed during this period. Surgical intervention was eventually performed, including tumor resection and placement of an interatrial patch. In the postoperative period, the patient developed right upper limb weakness and seizures. Brain imaging revealed an intraparenchymal hematoma in the left frontoparietal region (2.4 × 1.8 × 1.6 cm) and a smaller hematoma in the left occipital lobe (0.8 × 0.6 cm). Conservative management led to partial recovery of motor function and complete seizure control. The patient was discharged on anticonvulsant therapy.

**Figure 2:**
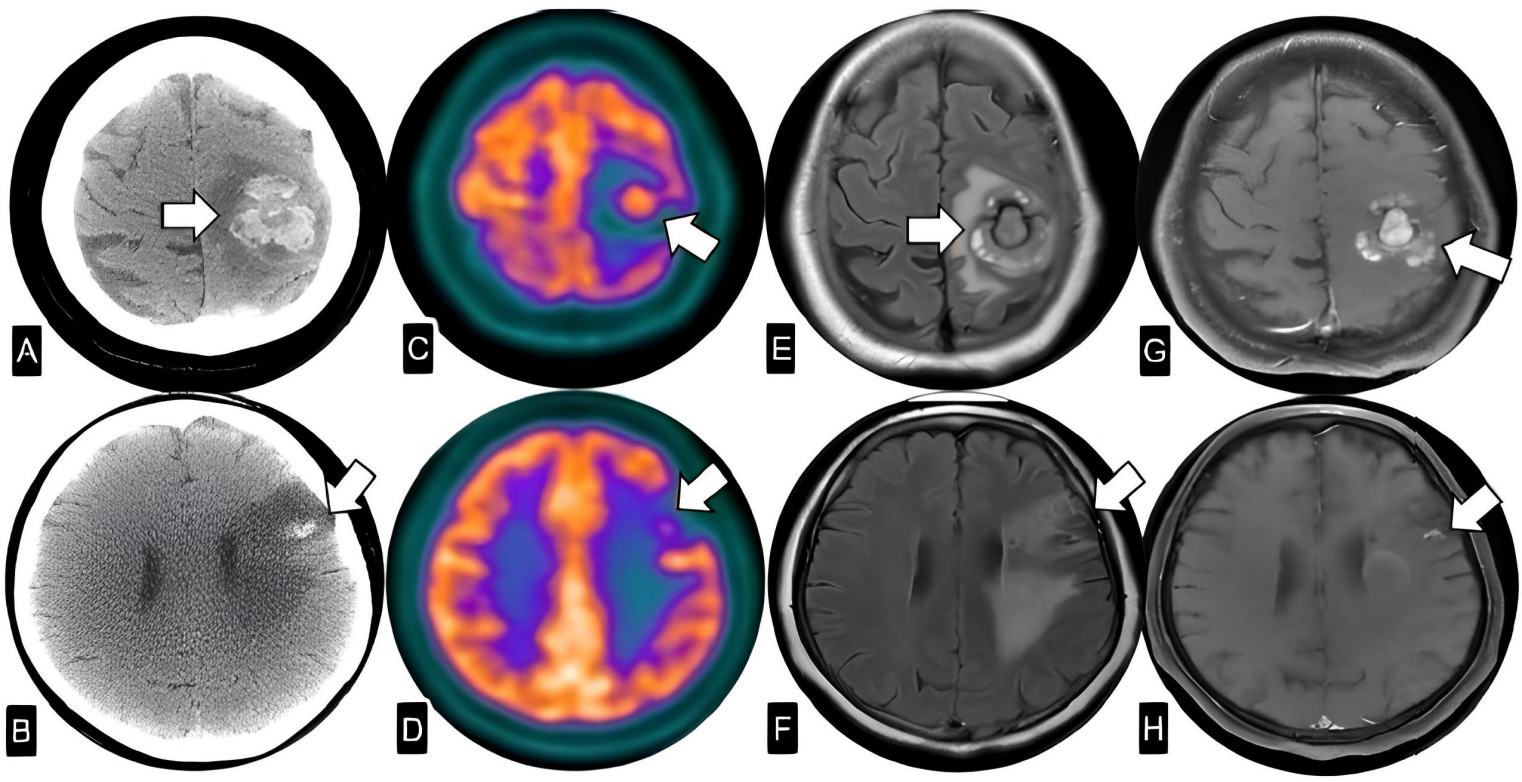
**(A, B)** Non-enhanced CT images. **(C, D)** Fused 18FDG-PET andCT images. **(E, F)** T2 FLAIR and **(G, H)** T1-Gd axial MRI. The images reveal two intraparenchymal expansive hemorrhagic and enhancing lesions in the left frontoparietal region with FDG uptake, suggestive of metastatic disease.

She was readmitted with worsening aphasia and new right-sided hypoesthesia. Imaging revealed a new hematoma in the left parietal lobe, enlargement of the pre-existing intraparenchymal hematomas, and subarachnoid hemorrhage. Further diagnostic workup included cerebral arteriography, which identified multiple fusiform aneurysms in distal branches of the right posterior cerebral artery and bilateral middle cerebral arteries. 18F-FDG PET/CT showed marked hypermetabolism in the left parietal hematoma (SUVmax: 5.2; late phase: 8.0) and moderate uptake in a second lesion in the left frontal operculum (SUVmax: 4.8; late phase: 5.5; **Figure 2**). Neither transesophageal echocardiography nor cardiac 18FDG-PET revealed evidence of a residual or recurrent cardiac myxoma.

At that time, differential diagnoses included hemorrhagic stroke secondary to vascular malformations, primary central nervous system neoplasms, or metastatic disease, however based on imaging findings suggestive of neoplastic involvement, an excisional biopsy was performed on the left frontoparietal lesion (measuring 0.3 × 2.0 × 0.8 cm). Histopathological and immunohistochemical analyses confirmed the diagnosis of a well-differentiated myxoid neoplasm. In light of the histopathological evidence, the hemorrhagic nature of the lesions, and the high recurrence risk associated with Carney Complex, the case was reviewed by a multidisciplinary tumor board for treatment planning. Radiotherapy was recommended to enhance local control and prevent further neurological decline. Volumetric modulated arc therapy (VMAT) was chosen over conventional whole-brain radiotherapy (WBRT) due to its superior dose modulation capabilities, enabling hippocampal sparing to preserve cognitive function in this young patient. VMAT allowed for the delivery of a total dose of 30 Gy to the whole brain, with a simultaneous integrated boost to 40 Gy targeting the lesions identified on 18F-FDG PET/CT, a regimen that was well-tolerated. Additionally, VMAT offered improved protection of intracranial vascular structures, a critical consideration given the presence of multiple fusiform aneurysms and the potential risk of radiation-induced vessel wall fragility.

One month after completing radiotherapy, the patient developed extensive deep vein thrombosis in the right lower limb. Due to her prior hemorrhagic strokes, anticoagulation therapy was contraindicated. She continued to experience moderate cognitive impairment, partial motor aphasia, and recurrence of cardiac myxoma.

**Figure 3:**
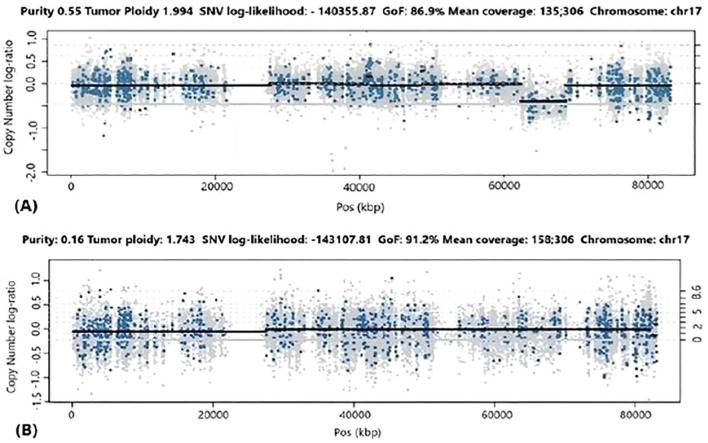
Analysis of Loss of Heterozygosity (LOH) and Copy Number Variation (CNV) on chromosome 17 using PureCN software. **(A)** Cardiac myxoma: the sample purity was 55%. The plot shows the log of the copy number ratio, indicating a copy number loss in the 17q24.2 region. **(B)** Cerebral metastasis: the sample purity was 16%. The plot shows the log of the copy number ratio, showing no evidence of LOH or CNV in the 17q24.2 region.

To explore a potential clonal relationship between the cardiac and intracranial tumors, we performed exome sequencing on both lesions. In the cardiac myxoma, somatic analysis revealed a loss of heterozygosity (LOH) at chromosome 17q24.2, encompassing the *PRKAR1A* gene. This alteration resulted in the loss of the wild-type allele, consistent with the tumor suppressor role of *PRKAR1A*. In contrast, no somatic variants or LOH events were identified in the intracranial lesion. However, this finding should be interpreted with caution, as the brain sample exhibited low tumor cellularity (16%), which limits the sensitivity of exome sequencing for detecting somatic or subclonal alterations. Therefore, the absence of detectable LOH does not definitively rule out a clonal relationship between the two tumors (**Figure 3**).

## 3 Discussion

The clinical manifestations of cardiac myxomas depend on tumor location, size, and morphology (solid or papillary). Left atrial tumors are most common (2) and may obstruct the mitral valve or embolize (7, 9). Constitutional symptoms such as fever, malaise, and weight loss are also frequent, likely mediated by tumor-derived interleukin-6 and other pro-inflammatory cytokines (9–11). Papillary myxomas, characterized by irregular and fragile surfaces, carry a particularly high risk of embolization. Embolic strokes affect 20%–30% of patients, with a predilection for the middle cerebral artery, as observed in our patient (7, 12), as observed in our patient. Women are more frequently affected, and 30%–50% of strokes involve multiple vascular territories (13–15).

Cerebral aneurysms, often fusiform and located in distal arterial branches, are common in patients with embolic myxomas. Although the underlying mechanism remains unclear, proposed hypotheses include occlusion by tumor emboli and subsequent invasion or proliferation within the intima or vasa vasorum, weakening the vessel wall (9, 16, 17). These aneurysms may appear before, during, or long after cardiac tumor diagnosis, displaying variable size and stability, sometimes regressing spontaneously. Most patients are under 60 years of age (18). Approximately 20% of patients with central nervous system (CNS) involvement experience subarachnoid and/or intraparenchymal hemorrhage (15, 19). This relatively low rate of hemorrhage may be due to dense connective tissue formed around the aneurysms, which may confer some protection, in contrast to other neoplastic aneurysms such as those seen in choriocarcinoma (19).

While embolic phenomena are well documented, true parenchymal implantation of myxoma cells is exceedingly rare. Only a limited number of cases describing such occurrences exist in the literature, each with heterogeneous clinical and radiological features (14, 16, 20–57) (**Table 2**). A comprehensive review by Chatzikonstantinou et al. identified 20 CNC patients with cerebral embolisms secondary to cardiac myxomas, none of whom demonstrated metastatic spread (58). When parenchymal involvement does occur, common symptoms include seizures, headaches, and hemiparesis (59–62). Affected individuals often share several features with patients harboring cardiac myxomas alone: a mean age of 30–40 years, female predominance, tumor origin in the interatrial septum’s oval fossa, and a mean tumor size of 4.6 ± 2.5 cm (59–62). Our patient exhibited many of these features, with the addition of recurrent myxomas, a hallmark of Carney Complex (63, 64).

**Table 2:** Reported cases of histopathologically confirmed cardiac myxoma with metastasis to the brain.^a^

Case	Year	Age/Sex	Initial symptom	Clinical Interval between Location of the disease	Interval between myxoma surgery and embolization	Location	Type/Size and site of atrial myxoma	Aneurysm formation	Other embolism	Other comorbidities	Treatment	Author
1	1978	44 F	Recurrent stroke	Hemiparesis Aphasia	+ 96 months	Choroid plexus	6×3×2 cm NA	NA	Bone	NA	Surgery	Rankin et al. (20) Seo et al. (21)
2*	1979	52 F	NAD	Death on arrival	Post-mortem	P, C, dura		NA	NA	NA	No	Budzilovichl et al. (22)
3*	1987	56 F	NAD	NAD	+ 48 months	C, Cerebrum		NA	NA	NA	Surgery	Bazin et al. (23)
4	1986	44 M	Recurrent stroke	Hemiparesis Seizures	+10 months	F-P	NA	NA	Skin	NA	Surgery	Morimoto et al. (24) Kadota et al. (25)
5	1990	54 M	NAD	Visual disturbances Seizures	+4 months	O	NA	NA		NA	Surgery	Ng HK (26)
6	1992	61 F	Fever, fatigue, dyspnea	Seizures	+8 months	F	NA	No		Malignant astrocytoma	Surgery	Chozick et al. (27)
7	1993	68 F	Hematuria	Headache, Visual disturbances	+11 months	O	3×3 cm	Yes	Kidney infarction	NA	Surgery	Chen et al. (28)
8	1993	70 M	Recurrent stroke	Hemiparesis	9 months before	P	Polypoid, 5×4×4cm	NA		NA	Surgery	Wada et al. (29) Kanda et al. (30)
9	1994	60 F	Seizures	Seizures	7 months before	P, dura	Polypoid, 5×4.5×2 cm Interatrial septum	Yes			Surgery	Samaratunga et al. (31)
10*	1997	64 M	NAD	Unspecific symptoms	+144 months	P-O	NA	NA	NA		Surgery	Scarpelli et al. (32)
11	2004	50 F	Hemiparesis	Hemiparesis	1 months before	P	Interatrial septum	NA			Surgery	Hirudayaraj et al. (33)
12	2005	41 F	Palpitation, Dyspnea	Seizures	+15 months	F-P-O, C	Large tumor	NA			Surgery + RT	Altundag et al. (34)
13	2005	30 F	Seizures	Hemiparesis, Seizures	+48 months	F-O	NA	Yes			NA	Balasuriya et al. (35)
14	2006	65 F	NAD	Hemiparesis, Seizures	+12 months	P-O	NA	NA		NA	Surgery	Rodrigues et al. (36)
15	2006	41 F	Paresthesia, Visual disturbance	Seizures, paresthesia	22 months before	F-P-O	Sessile 0.7×0.4 cm	Yes	Bone		Surgery	Rodriguez et al. (37) Lee et al. (38)
16	2007	35 M	Seizures	Seizures	48 months before	F-P-T-O	NA	NA		NA	Surgery + RT	Moiyadi et al. (39)
17*	2007	53 F	Seizures	Seizures	+48 months	F-P-O, C	NA	Yes		NA	Surgery	Rabarijaona et al. (40)
18	2008	60 M	Dizzness Palpitations, Fatigue	Seizures, Hemiparesis	+9 months	P-O, C	5.5×4×2 cm	No		NA	Surgery	Wolf et al. (41)
19*	2008	68 M	Stroke	Headache	+6 months	Multiple	NAD	NAD	NA	NA	Surgery + RT	Suzuki et al. (42)
20	2010	18 M	Dyspnea	Headache	+4 months	O	NA	Yes		BFAS	Surgery	Eddleman et al. (43)
21	2011	30 F	Seizures	Seizures	+24 months	F-P-O	5×5 cm	Yes			NA	Kumar et al. (44)
22	2012	15 F	NA	Headache	+44 months	P-O	NA	NA		NA	Surgery	Badrisyah et al. (45)
23	2012	45 M	NA	Seizures, hemiparesis	+18 months	F-P	NA	Yes		NA	Surgery	Radoi et al. (46)
24	2013	42 F	Amaurosis	Amaurosis	Simultaneous	F-O	NA	NA		NA	Surgery	Rique et al. (47)
25	2015	46 F	Seizures	Seizure	+24 months	F-P	Interatrial septum	No			Surgery	Côté et al. (48)
26	2015	41 F	Weakness and headache	Weakness and headache	Simultaneous	F	NA	Yes		NA	Surgery	Brinjikji et al. (14)
27	2015	34 F	Stroke	Headache	+ 24 month	T	NA	Yes		NA	Surgery	Brinjiki et al. (14)
28	2016	40 F	Dyspnea, Palpitations	Headache	10 days before	F-P-O, C	Large, exophytic 6×3.7× 3 cm	Yes		NA	Surgery + RT	Castaño et al. (49)
29	2017	41 F	Headache, Hemiparesis	Headache, hemiparesis	24 months before	F	Sessile Small	NA	Bone	NA	NA	El Sabbagh et al. (50)
30	2017	43 M	Stroke	Seizure	+9 months	P	irregular surface 3.2 × 4.3 cm	NA		NA	Surgery + RT	Ryu et al. (51)
31	2019	39 F	NAD	Headache, visual disturbances	+7 months	F-P-O	NA	Yes		NA	Surgery	Wan et al. (16) Zhang et al. (52)
32	2019	48 F	Headache	Headache	5 months before	F-P-O	Papillar 3.5×2.5×2.5 cm	Yes		Ovarian Cystadenomas	RT	Roque et al. (53)
33	2019	49 F	NAD	Headache	+11 months	P-O	NA	Yes		NA	NA	Zhang et al. (52)
34	2019	16 F	NAD	Hemiparesis	+34 months	P-O	NA	Yes		NA	NA	
35	2019	39 F	NAD	Hemiparesis	+8 months	P-O	NA	Yes		NA	NA	
36	2019	52 M	NAD	Seizure	+16 months	NA	NA	No		NA	NA	
37	2019	44 F	NAD	Hemiparesis	+1 months	NA	NA	No		NA	NA	
38	2020	62 M	Hemiparesis	Hemiparesis Blurred vision	+12 months	F-P-O	4.2×2.4 cm	NA		HPB, HCM (SLL/CLL)^b^ myxoma Cutaneous	Surgery	Maas et al. (54)
39	2020	63 M	Stroke	Seizures	+9 months	P-O	7×3 cm, interatrial septum	Yes		NAD	RT	Panos et al. (55)
40	2020	63 M	NAD	Headache	+12 months	P-O	NA	NA	NA	NA	Surgery	Ghodasara et al. (56)
41	2021	69 F	Recurrent stroke	Seizures, Distal tremor	+10 months	F-P-O, C	NA	NA		NA	Surgery	Aguilar et al. (57)
42	2025	46 F	Recurrent stroke	Seizures hemihypoesthesia	+3 months	P-F-O	3.3×2.5×1.5 cm interatrial septum	Yes		Carney Complex	Surgery + RT	Kuhn et al. (Present case)

F, female; M, male; P, parietal; F, Frontal; O, Occipital; C, cerebellum; T, temporal; NA, not available; BFAS, Bilateral femoral artery stenoses; HBP, high blood pressure; HCM, hypertrophic cardiomyopathy; SLL/CLL small lymphocytic lymphoma/chronic lymphocytic leukemia; RT, radiotherapy.

^a^Adapted from Aguilar et al., 2021 (57); added Brinjikji (14) and Ghodasara et al. (56).

^b^Histopathologic examination confirmed cardiac myxoma metastases and a small lymphocytic infiltrate within the tumor consistent with CLL/SLL.

^*^The data from (22, 23, 32, 40, 42) were not available in full text.

In most documented cases, brain lesions emerge years after cardiac myxoma resection, with a mean interval of 23.5 months (range: 1–144 months). Interestingly, in approximately 20% of patients, CNS lesions were identified before the cardiac tumor was diagnosed, with a mean lead time of 16.6 months (range: 0.3–48 months) (29–31, 33, 37–39, 49, 50, 53). Simultaneous detection of both conditions is extremely rare and has been reported in only two cases (14, 47).

Surgical resection is the mainstay of treatment for brain. Five cases have combined surgery with radiotherapy, as in our case (34, 39, 42, 49, 51), while only two were treated with radiotherapy alone (53, 55). Although there are concerns regarding the risk of aneurysm rupture after radiotherapy, due to potential weakening of the vessel wall, no hemorrhagic complications were reported among the three patients with aneurysms who underwent radiation therapy (39, 53, 55).

Unlike previous reports that used whole-brain radiotherapy (34, 39, 49, 51, 55), we opted for volumetric modulated arc therapy (VMAT) which allowed for lesion-specific dose boosting and hippocampal sparing, aiming to preserve cognitive function. This approach also limited radiation exposure to intracranial vascular structures, important given the presence of multiple fusiform aneurysms and concerns about radiation-induced vessel wall fragility.

In this case, histopathological analysis confirmed a well-differentiated myxoid neoplasm in the brain, and FDG-PET revealed hypermetabolism with elevated SUVmax values, supporting active neoplastic involvement. These findings reinforce the hypothesis of tumor cell dissemination, likely via embolic pathways, rather than conventional metastatic mechanisms. This contrasts with typical brain metastases, which often involve disruption of the blood-brain barrier by genes that promote migration, extracellular matrix degradation, and vascular permeability (65).

Notably, pathogenic variants in *PRKAR1A* are not commonly linked to brain metastases (65). A single previous case described molecular alterations in myxoid tissue without germline *PRKAR1A* mutations, identifying two variants of uncertain significance and two likely pathogenic variants in both cardiac and brain tissue, along with a copy number amplification at the *PRKAR1A* locus. Although gene amplifications typically involve oncogenes, it was hypothesized that the amplified allele harbored deleterious mutations, potentially explaining the tumorigenesis in that patient (53).

In contrast, our case presents a confirmed clinical and molecular diagnosis of Carney Complex, with two distinct hits in *PRKAR1A*: a germline pathogenic variant and loss of heterozygosity in the cardiac tumor, consistent with the classic tumor suppressor model. Although we could not assess somatic events in the brain lesion due to low cellularity, this appears to be the first reported case integrating histological, imaging, and genetic data to support a myxomatous brain lesion derived from cardiac origin in a Carney Complex patient. We propose that cardiac myxoma cells can, under specific conditions, disseminate through embolic routes, survive in distal tissues, and proliferate locally, ultimately forming tumors. This case illustrates how histologically benign neoplasms may display aggressive behavior, including distant colonization, particularly in the context of an underlying genetic predisposition.

## 4 Conclusion

This case highlights the rare but clinically significant complications of cardiac myxomas in Carney Complex, demonstrating that these histologically benign tumors can, under certain circumstances, exhibit aggressive behavior. It underscores the importance of maintaining a high index of suspicion for central nervous system involvement in patients with a history of left atrial myxomas who present with recurrent or unexplained neurological symptoms. The findings support the hypothesis that embolic dissemination may contribute to rare instances of myxomatous brain involvement, expanding our understanding of myxoma pathophysiology. Moreover, this case reinforces the value of integrated molecular and histopathological analysis in elucidating mechanisms of atypical tumor spread. Effective management of such complex presentations requires a multidisciplinary approach encompassing cardiology, neurology, oncology, genetics, and pathology to deliver personalized care.

## Data Availability

The datasets presented in this study can be found in online repositories. The names of the repository/repositories and accession number(s) can be found in the article/supplementary material.
